# Fecal supernatants from dogs with idiopathic epilepsy activate enteric neurons

**DOI:** 10.3389/fnins.2024.1281840

**Published:** 2024-01-31

**Authors:** Kristin Elfers, Antja Watanangura, Pascal Hoffmann, Jan S. Suchodolski, Mohammad R. Khattab, Rachel Pilla, Sebastian Meller, Holger A. Volk, Gemma Mazzuoli-Weber

**Affiliations:** ^1^Institute for Physiology and Cell Biology, University of Veterinary Medicine Hannover Foundation, Hannover, Germany; ^2^Department of Small Animal Medicine and Surgery, University of Veterinary Medicine Hannover Foundation, Hannover, Germany; ^3^Center for Systems Neuroscience (ZSN), Hannover, Germany; ^4^Veterinary Research and Academic Service, Faculty of Veterinary Medicine, Kasetsart University, Kamphaeng Saen, Nakhon Pathom, Thailand; ^5^Gastrointestinal Laboratory, Department of Small Animal Clinical Sciences, Texas A&M University, College Station, TX, United States

**Keywords:** fecal supernatant, canine idiopathic epilepsy, myenteric neurons, enteric nervous system, microbiota-gut-brain axis, phenobarbital, short-chain fatty acids

## Abstract

**Introduction:**

Alterations in the composition and function of the gut microbiome have been reported in idiopathic epilepsy (IE), however, interactions of gut microbes with the enteric nervous system (ENS) in this context require further study. This pilot study examined how gastrointestinal microbiota (GIM), their metabolites, and nutrients contained in intestinal contents communicate with the ENS.

**Methods:**

Fecal supernatants (FS) from healthy dogs and dogs with IE, including drug-naïve, phenobarbital (PB) responsive, and PB non-responsive dogs, were applied to cultured myenteric neurons to test their activation using voltage-sensitive dye neuroimaging. Additionally, the concentrations of short-chain fatty acids (SCFAs) in the FS were quantified.

**Results:**

Our findings indicate that FS from all examined groups elicited neuronal activation. Notably, FS from PB non-responsive dogs with IE induced action potential discharge in a higher proportion of enteric neurons compared to healthy controls, which exhibited the lowest burst frequency overall. Furthermore, the highest burst frequency in enteric neurons was observed upon exposure to FS from drug-naïve dogs with IE. This frequency was significantly higher compared to that observed in PB non-responsive dogs with IE and showed a tendency to surpass that of healthy controls.

**Discussion:**

Although observed disparities in SCFA concentrations across the various FS samples might be associated with the induced neuronal activity, a direct correlation remains elusive at this point. The obtained results hint at an involvement of the ENS in canine IE and set the basis for future studies.

## 1 Introduction

Epilepsy is the most common chronic neurological brain disease in dogs, as in humans (Berendt et al., 2015; Gesche et al., 2020). A diagnosis of idiopathic epilepsy (IE) often necessitates lifelong administration of anti-seizure medications (Bhatti et al., 2015). Among the array of available drugs, phenobarbital (PB) emerges as the most commonly prescribed first-line treatment for IE in dogs, owing to its proven efficacy and extensive clinical track record (Charalambous et al., 2014). Nevertheless, a subset of dogs does not respond favorably to PB therapy, continuing to experience epileptic seizures – a clinical phenomenon termed ‘PB resistance’ (Potschka et al., 2015).

Emerging scientific evidence suggests that gastrointestinal microbiota (GIM) may influence neurological conditions, including idiopathic generalized and focal epilepsy in humans through the microbiota-gut-brain axis (MGBA) (Yue et al., 2022). Notably, dogs with IE exhibit distinct GIM profiles compared to their healthy counterparts, characterized by diminished levels of gamma-aminobutyric acid (GABA) and bacteria that produce short-chain fatty acids (SCFAs) (Garcia-Belenguer et al., 2021). In a prior study, we demonstrated elevated concentrations of SCFAs produced by GIM in fecal samples following PB treatment. Butyrate levels were notably higher in PB-responsive dogs with IE than in their PB-non-responsive counterparts (Watanangura et al., 2022). Moreover, fecal microbiota transplantation, − a technique that involves transferring microbiota from a donor into a recipient’s gastrointestinal tract, has been proven to influence seizure susceptibility and duration of electrically induced seizures in rodent seizure models (Medel-Matus et al., 2018; Olson et al., 2018; Mengoni et al., 2021). Additionally, a recent study showed that gut microbiota-derived metabolites, including SCFAs, are present in the serum of human patients who have received fecal microbiota transplantation and that these metabolites are able to directly affect primary cultured microglia (Churchward et al., 2023). The pathways by which GIM and their products influence the brain after fecal microbiota transplantation remain unclear. Presently, there is a growing suspicion that these mechanisms may operate via neural pathways, notably emphasizing the role of the vagal nerve and the enteric nervous system (ENS).

The ENS consists of an extensive network of neurons and glial cells located within the wall of the whole gastrointestinal tract. It is composed of two ganglionated plexuses, the myenteric and the submucosal plexus and it harbors around 400 million neurons, which modulate intestinal functions independently from the central nervous system (CNS) (Wood, 1994; Goyal and Hirano, 1996; Gershon, 1999; Furness et al., 2014; Spencer and Hu, 2020).

A primary product resulting from the carbohydrate fermentation by GIM is SCFAs (Pilla and Suchodolski, 2021). These provide an energy source for the enterocytes and exhibit anti-inflammatory, immunomodulatory, and neuroprotective properties (Roediger, 1982; Andoh et al., 1999; Valvassori et al., 2016; Sadler et al., 2020). GIM are also associated with the synthesis of neurotransmitters and their precursors including glutamate and GABA, the main excitatory and inhibitory central neurotransmitters, respectively (Chen et al., 2021). Consequently, alterations in the GIM might impact the production of microbial SCFAs, potentially triggering inflammation. Moreover, these changes could disrupt the balance of neurotransmitters in the brain, either directly or indirectly by modulating vagal nerve activity. Such modulation might, in turn, influence the epileptic seizure threshold within the brain (Karl et al., 2017; Fülling et al., 2019). In recent years, GIM have garnered increasing attention due to their pivotal role in influencing the intensity, duration, and onset of therapeutic drug effects. For instance, they enhance the efficacy of immunotherapy in cancer treatment (Zhang et al., 2023) and modulate the anti-seizure effect of commonly used of anti-epileptic drugs (Thai et al., 2023).

The relationship between GIM, especially their microbial products like SCFAs, and IE warrants deeper exploration. Understanding this link could significantly influence the effective management of IE. Within the MGBA, signaling from the gut to the brain includes gastrointestinal hormones, tryptophan, the host’s immune and inflammatory system, microbial metabolites, and neurotransmitters. Moreover, the ENS and the vagal nerve may directly participate in signal transduction to the CNS (Fülling et al., 2019; Darch and McCafferty, 2022).

In this pilot study, we used fecal supernatants (FS) representing fecal contents from healthy dogs and dogs with IE, either drug-naïve, responsive, or non-responsive to PB. We measured SCFA concentrations in FS and applied FS directly onto primary cultured myenteric neurons from the guinea pig small intestine. The objectives of the study were to understand (1) the potential of the FS to induce neuronal responses, (2) to evaluate whether evoked neuronal activity differed based on the dogs’ IE treatment history and response to treatment, and (3) to measure the SCFA concentrations and composition in the different FS with regard to the dogs’ group assignment.

## 2 Materials and methods

This study is an in vitro experimental study.

### 2.1 Fecal samples

Fecal samples were collected from (A) healthy dogs as a control group, (B) drug-naïve, (C) PB responsive, and (D) PB non-responsive dogs with IE at the Department of Small Animal Medicine and Surgery of the University of Veterinary Medicine Hannover, Hannover, Germany. Each group contained six dogs of varying breeds, age, and sex (Table 1). Group A consisted of clinically healthy dogs that had not received medication, had not been vaccinated or dewormed during the previous 3 months prior to fecal sample collection. Groups B, C, and D included client-owned dogs with IE Tier II confidence level (De Risio et al., 2015). The dogs received the appropriate standard of care for IE management. Drug-naïve dogs allocated to group B had not yet received any anti-seizure drug. PB responsive dogs (group C) had been treated with PB for 3 months and had been seizure-free since the initiation of PB treatment. In this study, the highest standard “epileptic seizure freedom” was used to classify PB responsive dogs (Potschka et al., 2015). PB non-responsive dogs (group D) had received PB for at least 3 months. These dogs did continue to have epileptic seizures and did not have seizure free periods longer than three times the interictal period prior to PB treatment (Potschka et al., 2015). In each group, dogs were only included if their diets had remained unchanged during the last 3 months. A fecal sample from each dog was collected and stored in a plastic tube (5 mL, 57 × 15.3 mm, polypropylene; Sarstedt AG & Co. KG, Nürmbrecht, Germany) at −80°C within an hour after defecation.

**Table 1:** The table provides information of the dogs contributing fecal samples in this study (group A – healthy dogs, group B – drug-naïve dogs with idiopathic epilepsy, group C – phenobarbital-responsive dogs with idiopathic epilepsy, and group D – phenobarbital non-responsive dogs with idiopathic epilepsy).

**Group**		**Case no.**	**Breed**	**Sex**	**Age (year)**	**Age of IE onset (year)**	**CS/SE before PB**	**SF before treatment**	**SF**	**Serum PB conc. (μg/mL)**	**FS PB conc. (μg/mL)**	**Nutrition**	
												**Diets**	**Treats**
**A**	Healthy dogs (control)	1	Crossbreed	Female	3.7	–	–	–	–	–	–	CDF from fish	Beef treats
		2	Nova Scotia Duck Tolling Retriever	Male	6.1	–	–	–	–	–	–	CDF from poultry	Beef, poultry, pork, horse, and fish treats
		3	Dalmatian	Female	1.9	–	–	–	–	–	–	CDF from pork and chicken	Beef, poultry, pork, lamb, and fish treats
		4	Crossbreed	Male	11.6	–	–	–	–	–	–	CDF from horse	Chicken treats
		5	Crossbreed	Female	3.2	–	–	–	–	–	–	CDF from chicken	Beef, poultry, pork, and horse treats
		6	Beagle	Female	8.4	–	–	–	–	–	–	CDF from chicken	Duck treats
**B**	Drug-naïve dogs with IE	1	Peruvian Hairless Dog	Male	0.7	0.7	None	–	1	–	–	CDF from chicken	Pork, chicken, and rabbits treats, F, V, M
		2	Australian Shepherd	Male	2.8	2.8	None	–	1	–	–	CDF from ostrich	V
		3	Rottweiler	Male	3.5	3.5	CS	–	0.67	–	–	CDF from duck	Not reported
		4	Crossbreed	Male	1.2	1.2	None	–	2	–	–	CDF from lamb	Not reported
		5	French Bulldog	Male	3.3	1.3	None	–	0.33	–	–	CDF from chicken	Not reported
		6	Dachshund	Female	0.7	0.5	CS	–	1	–	–	CDF + CCF from duck	Beef treats, M
**C**	PB-responsive dogs with IE	1	Peruvian Hairless Dog	Male	1.0	0.7	None	2	0	14.7	3.50	CDF from chicken	Pork, chicken, and rabbits treats, F, V, M
		2	Australian Shepherd	Male	3.1	2.8	None	1	0	17.0	3.50	CDF from ostrich	V
		3	Crossbreed	Male	1.5	1.2	None	2	0	15.4	2.97	CDF from lamb	Not reported
		4	Dachshund	Female	1.0	0.5	CS	3	0	17.5	3.30	CDF + CCF from duck	Beef treats, M
		5	Rhodesian Ridgeback	Female	3.9	3.6	CS	3	0	17.6	4.21	CDF from duck, lamb, and horse	Pork, beef, and horse treats
		6	Australian Shepherd	Male	7.0	3.9	None	1	0	23.3	3.43	CDF from ostrich	V
**D**	PB non-responsive dogs with IE	1	Poodle	Male	6.9	6.2	SE	2	0.33	24.0	3.83	CDF from beef	F, V
		2	Labrador Retriever	Male	1.7	0.9	CS	4	4.3	34.2	3.33	CDF from chicken	Not reported
		3	Crossbreed	Male	7.8	0.7	CS	2	0.33	17.3	3.33	CDF from fish	Beef and duck treats, F, M
		4	French Bulldog	Male	3.6	1.3	None	1	0.33	19.2	3.61	CDF from chicken	Not reported
		5	Crossbreed	Female	6.7	5.8	None	2	1.33	21.6	3.45	CDF from chicken	Not reported
		6	Samoyed	Female	2.5	2.2	None	3	2.33	17.1	4.24	CDF + CCF from chicken	Chicken treats, V

Each group consists of six dogs. Fecal samples of these dogs were collected for this study. Signalment, age of epilepsy onset, experience of cluster seizure or status epilepticus, average seizure frequency before phenobarbital treatment and seizure frequency per month in the previous 3 months, serum and fecal supernatant phenobarbital concentrations, diets, and treats are shown. CS, cluster seizure; SE, status epilepticus; SF, seizure frequency; FS, fecal supernatant; PB, phenobarbital; conc., concentration; IE, idiopathic epilepsy; CDF, commercial dry food; CCF, commercial canned food; F, fruits; V, vegetables; M, milk products such as cheese and yogurt.

### 2.2 Fecal supernatants preparation

The fecal samples were thawed on ice. Then, 0.5 g feces from each sample was dissolved with 4 mL HEPES buffer [pH = 7.4, containing (in mM): 1 MgCl_2_, 1.25 CaCl_2,_ 1.2 NaH_2_ PO_4_, 135 NaCl, 5.4 KCl, 12.2 glucose, 3 HEPES] in a 15 mL Falcon tube using a plastic stick. The mixture was vortexed for 10 min before being centrifuged at 4°C, 10 min, 3,220 × g. The supernatant was removed using a 1 mL syringe and filtered with a 0.2 μm sterile syringe filter (VWR International, GmbH, Darmstadt, Germany). Aliquots of filtered supernatants were stored at −80°C until further use in neuroimaging experiments.

### 2.3 Serum and fecal phenobarbital concentration

The blood collections in both PB treated groups (groups C and D) were taken for clinical routine follow-up (Bhatti et al., 2015) on the same day of fecal sample collection. The concentrations of PB in serum and FS of group C and D were tested by Laboklin GmbH & Co KG, Kissingen, Germany.

### 2.4 Preparation of phenobarbital solution

To test for a direct effect of PB on cultured myenteric neurons, a solution was prepared containing a PB concentration comparable to the average PB value measured in FS from dogs in groups C and D (3.5 μg/mL). For this purpose, the oral form of a PB tablet (Luminaletten^®^, Virbac) was powdered, mixed with HEPES buffer to a final concentration of 3.5 μg/mL, and filtrated with a 0.2 μm sterile syringe filter.

### 2.5 Primary culture of guinea pig myenteric neurons

For cell culture experiments, intestinal tissue from n = 19 adult Dunkin Hartley guinea pigs of both sexes, weighing approximately 300–400 g (10–12 weeks of age) was used. Intestinal tissues from guinea pigs were chosen because their intestines are well-characterized and provide an ideal and straightforward preparation for studying enteric neurons in mammals (Brookes, 2001). After stunning the animals using a spring-loaded stunning device, followed by exsanguination, small intestinal tissue was removed to obtain primary culture of enteric neurons as described elsewhere (Kugler et al., 2015, 2018; Elfers et al., 2023). The methods used for stunning and killing of the guinea pigs were in accordance with Annex IV of Directive 2010/63/EU and the German Animal Welfare Act and have been approved by the Animal Welfare commissioner of the University of Veterinary Medicine Hannover, Foundation, Hannover Germany (approval number: TiHo-T-2022-5). Briefly, longitudinal muscle/myenteric plexus preparations (LMMP) were separated by stripping the plexus together with longitudinal muscle and serosa with a fine pair of forceps. The LMMPs were cut into small pieces (1 × 1 mm), enzymatically digested, and 200 μl of the myenteric ganglia suspension was seeded in cell culture dishes (Ibidi GmbH, Martinsried, Germany). This was incubated in medium M199 supplemented with 10% fetal bovine serum (FBS) (Gibco™), 50–100 ng mL^−1^ mouse nerve growth factor 7S (Alomone Labs, Jerusalem, Israel), 5 mg mL^−1^ Glucose, 100 U mL^−1^ Penicillin, 100 mg mL^−1^ Streptomycin (Gibco™), and 2 mM arabinose-C-furanoside (Sigma-Aldrich Corporation). The neurons were cultured in vitro under standard culture conditions (5% CO_2_; 37°C; humidity 95%) for at least 11–14 days to obtain interconnected ganglion-like, neuronal clusters. The medium with additives was changed every 2–3 days. For the neuroimaging recordings, dishes were placed in a homemade culture dish holder and continuously perfused with 37°C Krebs solution (for composition; see below).

### 2.6 Neuroimaging with voltage sensitive dye

Electrical signals in cultured myenteric neurons were detected with an ultrafast neuroimaging technique with the fluorescent voltage-sensitive dye 1-(3-sulfanato-propyl)-4-[b-[2-(di-n-octylamino)-6-naphtyl] vinyl] pyridinium betaine (Di-8-ANEPPS, Thermo Fisher Scientific) (Kugler et al., 2015, 2018; Elfers et al., 2023). Cultured neurons were stained with Di-8-ANEPPS (20 μM) by 12 min incubation at room temperature in the dark (Kugler et al., 2015, 2018; Elfers et al., 2023). Afterwards, the dishes were mounted on an inverted epifluorescence microscope (Olympus IX71; Olympus Corporation, Hamburg, Germany) and were continuously superfused with 37°C Krebs solution (pH = 7.4) containing (in mM): 1.2 MgCl_2_, 2.5 CaCl_2,_ 117 NaCl, 15 NaHCO_3_, 4.7 KCl, and 11 Glucose. To detect neuronal signals, the cultures were excited with a light emitted by a green high-power LED (LET A2A true green (521 nm) 700 mA; OSRAM GmbH, Munich, Germany) in combination with a filter-set containing a 525/15 nm bandpass excitation filter (AHF Analysentechnik AG Tübingen, Germany), a dichroic mirror with a separation wavelength of 565 nm, and a bandpass filter with a spectrum of 560/15 nm (AHF Analysentechnik AG). Using a 40× oil immersion objective lens (UApo 40× OI3/340 Oil NA 1.35–0.5; Olympus Corporation), the required high light intensity and appropriate signal-to-noise ratio were achieved. Changes in Di-8-ANEPPS fluorescence intensity were detected by a high-speed complementary metal oxide semiconductor (CMOS) camera with a 1.25 kHz frame rate and a spatial resolution of 256 × 256 pixels (DaVinci1K, RedShirt Imaging, LLC, Decatur, GA, USA). Combined with the 40x oil immersion objective lens, this resulted in a spatial resolution of 2.2 μm^2^ per pixel. Generated optical data were analyzed using the TurboSM 64 software (RedShirt Imaging LLC^1^). FS were thawed at 4°C shortly before they were applied directly onto single neuronal clusters by 500 ms of local pressure application (PDES-2lL; npi electronic GmbH, Tamm, Germany). Each supernatant was applied to at least 6–10 clusters (dependent on the number of clusters present in each culture dish and with cluster defined as an accumulation of at least three neurons in close proximity) in two distinct dishes from one or more different cultures. The application was performed with a delay of 200 ms in order to record potential sponateous neuronal activity and distinguish it from FS-elicited activity. In a different set of experiments, a PB solution containing 3.5 μg/mL PB was applied directly onto single neuronal clusters in the same way as described above in order to test for a potential direct neuronal excitation by PB.

### 2.7 Immunohistochemistry

Staining for neuron-specific enolase (NSE) was used to count total numbers of neurons per cluster. The cultured neurons were fixed immediately after the neuroimaging experiments for 15 min at room temperature in a solution containing 4% paraformaldehyde and 0.002% picric acid (Sigma-Aldrich Corporation). Afterwards, tissues were washed (3 × 10 min) in phosphate-buffer saline (PBS) and pre-incubated for 1 h in PBS containing 4% horse serum (Sigma-Aldrich Corporation) and 0.5% Triton X-100 (Sigma-Aldrich Corporation). The following cultures were incubated for 12 h at room temperature in a solution containing the primary antibody (rabbit anti-NSE, 1:8000, Polysciences, Inc., Warrington, PA, USA) and then washed three times in PBS and incubated for 2 h in a solution containing the secondary antibody (Cy3-conjugated donkey anti-rabbit IgG 1:500 from Dianova GmbH, Hamburg, Germany). In a final step, cultures were washed three times in PBS and covered with a solution of PBS (pH 7.0) containing 0.1% NaN_3_ and 80% glycerol (Sigma-Aldrich Corporation). Cultures were examined using an epifluorescence microscope and appropriate filters. Images were taken and analyzed with a monochrome camera (XM 10; Olympus Corporation) combined with Olympus cellSens Standard Software (Olympus Corporation^2^).

### 2.8 Short-chain fatty acid analysis

The SCFA concentrations in FS, including acetate, propionate, butyrate, isobutyrate, valerate, and isovalerate were measured using a stable isotope dilution gas chromatography–mass spectrometry (GC–MS) assay. The FS samples were stored at −80°C until analysis. After thawing, samples were vortexed briefly, 500 μL of supernatant was mixed with 25 μL of internal standard (200 mM heptadeuterated butyric acid) and extracted using a C18 solid phase extraction column (Sep-Pak Vac C18 1 cc/100 mg Cartridge, Waters Corporation, Milford, MA, USA). Samples were derivatized using 50 μL N-tert-butyldimethylsilyl-N-methyltrifluoroacetamide (MTBSTFA) and incubated at room temperature for 60 min. A GC (Agilent 6,890 N, Agilent Technologies Inc., Santa Clara, CA, USA) coupled with an electron ionization MS (Agilent 7890A, Agilent Technologies Inc.) was used for chromatographic separation and quantification of the derivatized samples. Separation was achieved using a DB-1 ms capillary column (Agilent Technologies Inc.). The GC temperature program was as follows: 40°C held for 0.1 min, increased to 70°C at 5°C/min, 70°C held for 3.5 min, increased to 160°C at 20°C/min, and finally increased to 280°C at 35°C/min, then held for 3 min. The total run time was 20.53 min. The MS was operated in electron impact positive-ion mode with selective ion monitoring at mass-to-charge ratios (M/Z) of 117 (acetate), 131 (propionate), 145 (butyrate and iso-butyrate), 152 (heptadeuterated butyrate; internal standard), and 159 (valerate and isovalerate). Quantification was based on the ratio of the area under the curve of the internal standard and each of the fatty acids (Moreau et al., 2003; Minamoto et al., 2019).

### 2.9 Data analysis and statistics

Raw data of neuroimaging experiments were analyzed using the TurboSM (RedShirt Imaging) and Igor Pro 6.22A (Wavemetrics Inc., Lake Oswego, OR, USA) software. Statistical evaluation and graphic display were performed using Excel 2016 (Microsoft Corporation, Redmond, WA, USA), Prism^®^ version 9.0.0 (GraphPad Software, LLC, San Diego, CA, USA), and ImageJ 1.8.0 (Wayne Rasband, National Institute of Health, Bethesda, MD, USA). Due to non-Gaussian distribution of most data (tested with the Shapiro–Wilk normality test), neuroimaging data are presented as the median and [Q_25_/Q_75_]. For neuroimaging experiments, we calculated the proportion of neurons per cluster (based on NSE positive immunoreactivity) responding to the application of PB or supernatants, the number of action potentials (APs) fired by each responding neuron, and their burst frequency, defined as number of APs divided by overall duration of spike discharge. Responders were defined as neurons, which started firing APs after (or during) the application of the FS and did not show spontaneous activity during the delay time before the FS application. Neuronal responses to direct PB application were compared to baseline neuronal activity without any stimulus (blank) by the Wilcoxon signed rank test. Activity from these blank recordings was compared to the neuronal responses after application of the different groups of FS with the Kruskal-Wallis test and Dunn’s multiple comparisons test. To test for significant differences of neuronal responses to the different groups of FS, the Kruskal-Wallis test, followed by Dunn’s multiple comparison test were applied. N numbers are given as numbers of animals (which equal the number of cultures, since every culture was derived from intestinal tissue from a single animal)/clusters, the particular FS or PB solution were applied on. The PB concentrations in serum and FS of groups C and D as well as the SCFA concentrations in FS of all groups were tested for normal distribution using the Shapiro–Wilk normality test. Due to the Gaussian distribution of PB concentrations in both groups and the non-Gaussian distribution of some SCFA data, further analyses were performed by one-way unpaired t-test and Kruskal–Wallis, followed by Dunn’s multiple comparison test. *p* < 0.05 was considered statistically significant. The SCFA concentrations are presented as means ± SD.

## 3 Results

### 3.1 Animals and phenobarbital concentration

Details of the study population and its individual PB concentrations can be found in Table 1. The age range and sex distribution for the dogs in each group were as follows: the healthy control group (group A) included dogs aged 1.9–11.6 years, consisting of 4 females and 2 males; the drug-naïve dogs with IE (group B) were aged 0.7–3.5 years, with 1 female and 5 males; the PB-responsive dogs with IE (group C) were aged 1–7 years, comprising 2 females and 4 males; and the PB non-responsive dogs with IE (group D) had ages ranging from 1.7 to 7.8 years, with 2 females and 4 males.

There were no significant differences in PB concentrations, neither in serum nor in FS between the group of PB-responsive dogs with IE and PB non-responsive dogs with IE.

### 3.2 Neuroimaging experiments: canine fecal supernatants excite primary myenteric neurons

**Figure 1:**
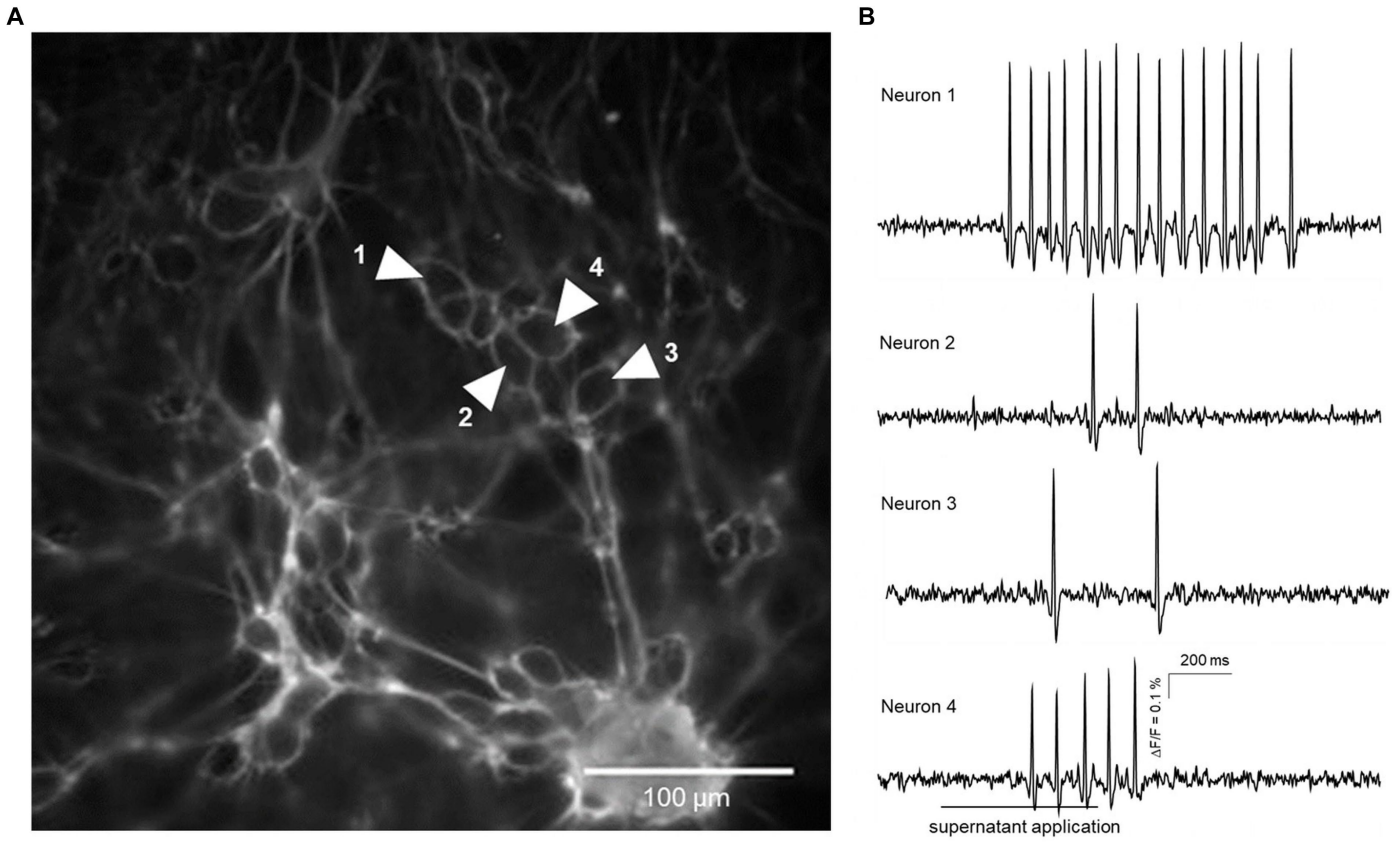
Action potential discharge in guinea pig primary myenteric neurons evoked by fecal supernatant application from a group B dog (drug-naïve with epilepsy). **(A)** shows a cluster of primary myenteric neurons cultured for 12 days with outlines of individual neurons labeled by Di-8-ANEPPS. Traces from four neurons marked by shaded white arrows are shown in **(B)**. Application of the fecal supernatant was delayed by 200 ms and done for 500 ms (indicated by the bar below the trace of neuron 4) and evoked action potential discharge in each of the four marked neurons.

**Figure 2:**
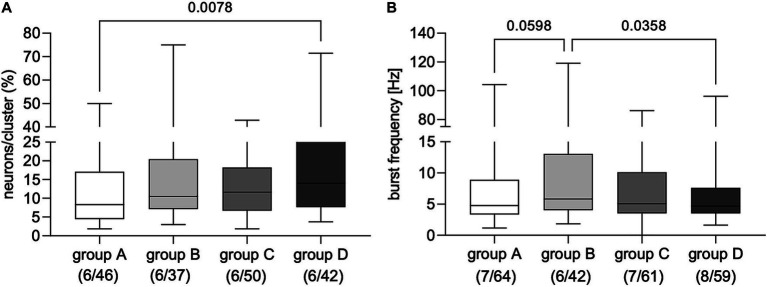
Proportion of primary myenteric neurons responding to application of fecal supernatants from healthy controls (group A) and dogs with idiopathic epilepsy (IE; groups C–D). **(A)** Application of fecal supernatants from phenobarbital (PB) non-responsive dogs with epilepsy (group D) evoked activity in a significantly greater proportion of primary myenteric neurons compared to supernatants from healthy controls assigned to group A (Kruskal–Wallis test: *p* = 0.02 with Dunn’s multiple comparison test). **(B)** Evoked neuronal burst frequency was significantly higher after application of fecal supernatants from drug-naïve dogs with IE (group B) than from PB non-responsive dogs with epilepsy from group D; a statistical trend was revealed for supernatants from healthy control dogs (group A) compared to those from drug-naïve dogs (group B); (Kruskal–Wallis test: *p* = 0.03 with Dunn’s multiple comparison test). Data shown are the medians with the 25th and 75th quartiles as a box plot and the minima and maxima as a whisker plot. N numbers (cultures/clusters) supernatants were applied on are given in parenthesis.

Applying FS from dogs with IE evoked AP discharge in a proportion of cultured myenteric neurons from guinea pigs (Figure 1 and Supplementary Figures S1–S3). Statistical comparison revealed that applying FS from non-responsive dogs with IE to PB treatment (group D) induced neuronal activity in a significantly greater proportion of enteric neurons than FS from healthy controls (group A; Figure 2A). However, calculated burst frequency was highest after FS application from drug-naïve dogs with IE (group B, 5.8 [4.1/13.1] Hz) and was significantly greater than the burst frequency after applying FS from group D dogs (4.7 [3.5/7.6] Hz, *p* = 0.04; Figure 2B). A statistical trend was revealed between evoked burst frequency induced by application of FS from group A (healthy controls; 4.8 [3.3/8.9] Hz, compared to group B, *p* = 0.06; Figure 2B). Application of FS from all dogs, regardless of group, induced comparable numbers of APs/neuron (group A: 3 [2/4], *n* = 7/64; group B: 3 [2/5], *n* = 6/42; group C: 3 [2/4], *n* = 7/61; group D: 3 [2/5], *n* = 8/59).

Neuronal activity in regard to burst frequency evoked by application of a 3.5 μg/mL PB solution did not differ from recorded non-stimulated baseline activity (baseline: 3.6 [2.8/5.0] Hz vs. PB: 3.5 [2.2/5.2] Hz, *n* = 3/31, *p* = 0.75, Wilcoxon test). Comparison of the non-stimulated baseline burst frequency with the FS-induced frequencies revealed significantly higher frequencies after FS application (Kruskal–Wallis test *p* = 0.0002, Dunn’s multiple comparisons test: blank vs. group A *p* = 0.02; blank vs. group B *p* < 0.0001; blank vs. group C *p* = 0.005; blank vs. group D *p* = 0.04, respectively).

### 3.3 Short-chain fatty acids concentrations in fecal supernatants

**Figure 3:**
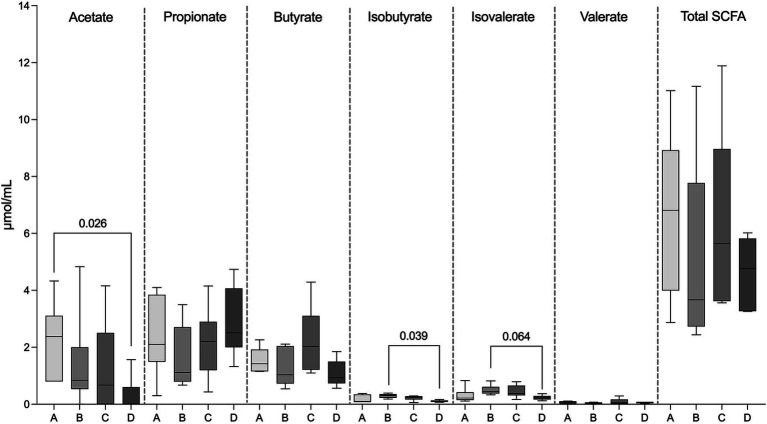
Concentration of SCFAs in the fecal samples, including acetate, propionate, butyrate, isobutyrate, isovalerate, valerate, and total SCFAs of fecal supernatants (FS) from healthy dogs (group A) and dogs with epilepsy either drug-naïve (group B), PB-responsive (group C), or PB non-responsive (group D). The acetate concentrations in FS from group A were significantly higher than those in FS from group D. The isobutyrate concentrations in FS from group B were significantly higher than those in group D, while isovalerate concentrations in FS from group B tended to be higher compared to those in group D. Data shown are the medians with 25th and 75th quartiles as a box plot and minima and maxima as whisker plot. *N* = 6 per group. (Kruskal–Wallis test with Dunn’s multiple comparison test).

Total SCFA concentrations as well as concentrations of propionate, butyrate, and valerate did not differ in FS between the four examined groups (*p* = 0.44). However, acetate concentrations were significantly higher in FS from the healthy control group (group A, 2.23 ± 1.34 μmol/mL) compared to the PB non-responsive group (group D, 0.31 ± 0.63 μmol/mL) (*p* = 0.03), while the concentrations of iso-butyrate (0.29 ± 0.08 μmol/mL) and isovalerate (0.49 ± 0.18 μmol/mL) in FS from the PB responsive group (group C) were significantly higher (*p* = 0.04) and tended to be higher compared to those in FS from the PB non-responsive group (group D, isobutyrate; 0.11 ± 0.03 μmol/mL, isovalerate; 0.23 ± 0.08 μmol/mL; *p* = 0.064) (Figure 3).

## 4 Discussion

The results from this study demonstrate the effects of directly applying FS from dogs – both with and without IE, whether treated with PB or drug-naïve – onto cultured myenteric neurons isolated from the guinea pig’s small intestine. The observed neuronal activity seen in enteric neurons upon exposure to FS substantiates our hypothesis regarding a connection between GIM and their derivatives with the ENS. Notably, both the FS from healthy dogs and dogs with IE were able to evoke activity in myenteric neurons. However, FS from PB non-responsive dogs activated the greatest proportion of enteric neurons, albeit with the lowest burst frequency. Additionally, an examination of the concentrations and composition of SCFAs in the different FS revealed a reduction in acetate and in branched SCFAs in FS from dogs with PB non-responsive IE. These specific SCFAs have previously been identified as potential energy sources, exhibiting neuroprotective properties, and possessing anti-inflammatory attributes (Roediger, 1982; Andoh et al., 1999; Valvassori et al., 2016; Sadler et al., 2020).

There are several substances in FS capable of directly activating enteric neurons (Xia et al., 1999; Neunlist and Schemann, 2014; Fung et al., 2021). These include, among others, dietary- (SCFAs) and drug- (PB) derived substances, as well as bile acids and inflammatory mediators (Prakash et al., 2013; Khan and Zaki, 2021; Zawadzki et al., 2022).

The modified production of SCFA by GIM in PB-treated dogs may partially account for the shift in recorded FS-induced enteric neuronal burst frequency, aligning them with values observed in the control group. A direct activation of enteric neurons by SCFA has been already reported in a study by Fung and colleagues (Fung et al., 2021). SCFAs have been shown to modulate enteric neuronal activity both, via the SCFA receptor GPR41 (Nøhr et al., 2013) and the SCFA transporter MCT2 (Soret et al., 2010). Both direct SCFA application onto enteric neurons, as well as diet-induced changes in SCFA production in rodent *in vivo* models modulated enteric neuronal activity and their phenotype and functions (Neunlist et al., 1999; Soret et al., 2010; Fung et al., 2021; Vicentini et al., 2021). Furthermore, the SCFA concentrations differed depending on the therapeutic status of the dogs with IE (Watanangura et al., 2022).

Though PB contained in the FS from dogs in group C and D would have been able to directly elicit enteric neuronal activity via GABA_A_ receptor-induced chloride efflux (Cherubini and North, 1984; Poulter et al., 1999; Krantis, 2000), this was not the case, as indicated by our results after applying a PB solution. Therefore, the recorded effects after FS application must be related to other compounds within FS or to PB’s metabolites. However, since the PB concentrations we measured in the FS in our study were relatively low, this could be a possible explanation for the missing direct effect on enteric neuronal activity in our study. The PB concentration in the FS could be influenced by its absorption and metabolization by the GIM and hence appeared to a minor amount in the collected fecal samples (Mukhtar et al., 2022).

Furthermore, the SCFA concentrations were different depending on the therapeutic status and response of dogs with PB treated IE. PB administration did increase fecal SCFAs’ concentrations in drug-naïve dogs with IE and showed a higher concentration of butyrate in PB responders compared to non-responders (Watanangura et al., 2022). It is reasonable to assume that SCFAs at least partly mediated the recorded differences in enteric neuronal activity in the current study. We found the same tendency for butyrate concentrations as well as for isobutyrate and isovalerate concentrations when comparing PB responsive group to PB-non-responsive group. Nonetheless, a greater sample size may provide more objective results, since the reference range of each SCFA is wide in domestic dogs (Minamoto et al., 2019). Our results indicate a decrease in the concentrations of branched SCFAs, which are derived from microbial amino acid degradation, to different extents in the FS from dogs with IE (groups B, C, D). The extent to which this metabolic pathway is affected may correlate with the specific therapeutic outcomes observed in these groups. This aligns with the emerging concept of a bidirectional relationship between GIM and the effectiveness of anti-seizure treatments, as recently demonstrated in a mouse seizure model (Thai et al., 2023). Nevertheless, the precise impact of observed changes in SCFA concentrations on the intestinal environment and consequent responses of the ENS remains speculative at present. Indeed, it has been shown that some enteric neurons can be stimulated by either acetate, propionate, butyrate, or a combination of at least two of them (Fung et al., 2021), while little is known about the effects of isobutyrate and isovalerate on enteric neurons. Information on synergistic or even antagonistic effect are lacking. Therefore, in future experiments on enteric neuronal activity, different amounts and proportions of these components should be considered.

Though not investigated in the current study, another component present in the FS probably to different extents depending on the health and treatment status of the dogs, are bile acids (BA). BA metabolism is affected by GIM and hence changes in the microbiota directly affect the fecal BA profile as also shown for dogs with intestinal diseases (Blake et al., 2019). As BA are able to directly activate enteric neurons (Poole et al., 2010), one could speculate that the BA profile in FS from dogs with IE differs based on their treatment history, providing a potential explanation for the differences in the recorded neuronal activity. Detailed BA profiles should be considered in future studies of GIM in canine epilepsy.

GIM changes could influence local inflammatory processes in the intestine, which potentially affect the CNS by sending GIM metabolites or signals from the intestine via the bloodstream or ENS through the vagal nerve (Hathaway et al., 1999; Riazi et al., 2010; Takeda et al., 2013). It has been reported that inflammation may mediate epileptogenesis (Rana and Musto, 2018). One study reported that dogs with drug-resistant IE had an elevated number of circulating T helper cells, indicative of an inflammatory response (Knebel et al., 2022). Human patients with drug-resistant epilepsy showed differences in GIM compared to healthy controls and drug-responsive patients with an elevated abundance of gram-negative bacteria (Peng et al., 2018; Safak et al., 2020; Gong et al., 2021), which increased the risk of inducing inflammatory processes by their lipopolysaccharide production (Lüderitz et al., 1982; Lively and Schlichter, 2018). Our previous study results revealed that the feces of PB non-responsive dogs contained lower concentrations of anti-inflammatory butyrate compared to PB-responsive dogs (Watanangura et al., 2022). Combined with the current results showing that FS from non-responders had also lower acetate concentrations compared to healthy controls, these findings could hint at a reduced SCFA-mediated anti-inflammatory effect, but a pro-inflammatory effect of altered fecal composition in non-responsive dogs (Xia et al., 1999). Physiologically, the crosstalk between intraluminal GIM metabolites and enteric neurons needs an intact mucosa layer for signal transduction (Fung et al., 2021). The inflammatory process could negatively affect the intestinal barrier and increase intestinal permeability, finally leading to an enhanced exposition of enteric neurons to luminal contents including GIM, their products, and cytokines, which could activate or even damage the neurons (Ding et al., 2021). In future studies, measurement of inflammatory mediators in FS could provide more information to this point.

Stimulation of the vagal nerve is an accepted management option for drug-resistant epilepsy (Pigarev et al., 2020). It has been shown that certain stimulation patterns significantly improve epileptic seizure frequency, with some patients becoming epileptic seizure free. Here, we report a change in enteric neuronal activation and burst patterns. Hypothetically, altered myenteric neuronal activity could also influence vagal nerve firing patterns, in line with the concept of an intramural sensory ENS to vagus transmission (Perez-Burgos et al., 2014) and therefore indirectly the brain, changing its susceptibility to seizure generation and propagation. However, this was not shown within the current study and needs further investigation. In addition, as previously mentioned, PB can potentially modulate neurons at elevated fecal concentrations and alter butyrate levels. These changes could influence enteric neuronal burst activity, representing an additional mechanism of action for anti-seizure drugs (Rho et al., 1996; Watanangura et al., 2022).

Some limitations of the current study need to be considered. Cultures of myenteric neurons were derived from healthy guinea pigs, not dogs, which could introduce species difference. However, the intestines of guinea pigs are well-studied and commonly used model to study gut physiology (Brookes, 2001; Gombash et al., 2017). Furthermore, the chosen method of primary (my-)enteric cell culture is an established method to study enteric neurons, particularly their electrophysiological and functional profiles, expression of distinct neuronal receptors, or neurotransmitter release and how neurons might be affected by different stimulants or compounds (Zhou et al., 2002; Smith et al., 2013; Brun and Akbarali, 2018; Elfers et al., 2023). The advantage of this method is that it provides an opportunity to study properties of the enteric neurons isolated from the context and to directly test drugs as therapeutic options aiming to modulate the electrical activity of such neurons. Another limitation is that there was no dietary standardization prior to fecal sample collection which could influence some metabolites of GIM (Schmidt et al., 2018; Pilla and Suchodolski, 2021), and dogs with IE were from a heterogeneous population, characterized by varying signalment and age of epilepsy onset. These differences may lead to minor variations in GIM and their products among individuals (Reddy et al., 2019; You and Kim, 2021). This should be considered when interpreting the results of the current study. It should be stressed that this is a pilot study and cannot yet be extrapolated to the entire canine IE population, especially as only a small number of dogs were included in each group. In addition, we focused on SCFA differences between groups, which does not necessarily mean that there are no other chemical compounds, which could explain differences between the groups. Future studies need to focus on identifying differences in composition of FS of the different groups and then study how the individual factors identified could influence ENS function. This might also include experiments using specific blockers of, e.g., SCFA receptors and transporters and as already mentioned investigation of differences among SCFAs, including branched and non-branched. The preliminary findings from this pilot study are promising, indicating that FS from dogs with IE can activate myenteric neurons to varying degrees based on their treatment status and responsiveness. These initial insights suggest the ENS may indeed have a role in canine IE. However, this can only be seen as one study followed by many to improve our understanding of how GIM, ENS, and CNS are interlinked. Future studies should include larger cohorts also considering and correcting for potential confounding factors such as age, sex and diet. Furthermore, the current findings are not sufficient to draw final conclusions or to directly relate them to therapeutic options.

## 5 Conclusion

The current pilot study showed that *in vitro*, enteric neurons derived from guinea pigs can be activated by components present in FS of healthy dogs and those with IE. The recorded differences in the induced neuronal activity by application of FS from the different groups might be associated with differences in the FS SCFA profile between healthy dogs and dogs with IE, though with the current experimental setup we were not able to directly show a potential link. The interplay between GIM and their metabolites, their signaling to enteric neurons, and the following signaling to the CNS via the MGBA might play a role in IE. Further studies are necessary to investigate which compounds in detail were responsible for the changes seen in the current pilot study. Furthermore, there is a need for studies of how local processes in the intestine of epileptic dogs and associated enteric neuronal activation might be communicated to the brain via systemic circulation or ascending neural pathways.

## Data availability statement

The original contributions presented in the study are included in the article/Supplementary material, further inquiries can be directed to the corresponding author.

## Ethics statement

Ethical approval was not required for the studies involving animals in accordance with the local legislation and institutional requirements because only fecal samples after defecation were collected to use in this study. Written informed consent was obtained from the owners for the participation of their animals in this study.

## Author contributions

KE: Conceptualization, Investigation, Methodology, Writing – original draft, Writing - review & editing. AW: Conceptualization, Investigation, Methodology, Writing – original draft, Writing - review & editing. PH: Investigation, Methodology, Writing – review & editing. JS: Investigation, Supervision, Writing – review & editing. MK: Investigation, Writing – review & editing. RP: Investigation, Writing – review & editing. SM: Conceptualization, Supervision, Writing – review & editing. HV: Conceptualization, Supervision, Writing – review & editing. GM-W: Writing – review & editing, Methodology, Supervision, Conceptualization.

## Footnotes

^1^ www.redshirtimaging.com

^2^ https://www.olympus-lifescience.com/de/software/cellsens
