# Supplementary material for: Fecal supernatants from dogs with idiopathic epilepsy activate enteric neurons
Source: Front Neurosci. 2024 Jan 31;18:1281840. doi: 10.3389/fnins.2024.1281840 (PMC10864448; doi:10.3389/fnins.2024.1281840)
Supplement: Supplementary file 1 [file Data_Sheet_1.PDF]

Supplementary Material

Fecal Supernatants from Dogs with Epilepsy Activate Enteric Neurons

Kristin Elfers[1,†], Antja Watanangura[2,3,4,*,†], Pascal Hoffmann[1], Jan S. Suchodolski[5], Mohammad R. Khattab[5], Rachel Pilla[5], Sebastian Meller[2], Holger A. Volk[2,3] and Gemma Mazzuoli-Weber[1,3]

*** Correspondence:** Antja Watanangura: antja.watanangura@tiho-hannover.de

Supplementary Figures

S1

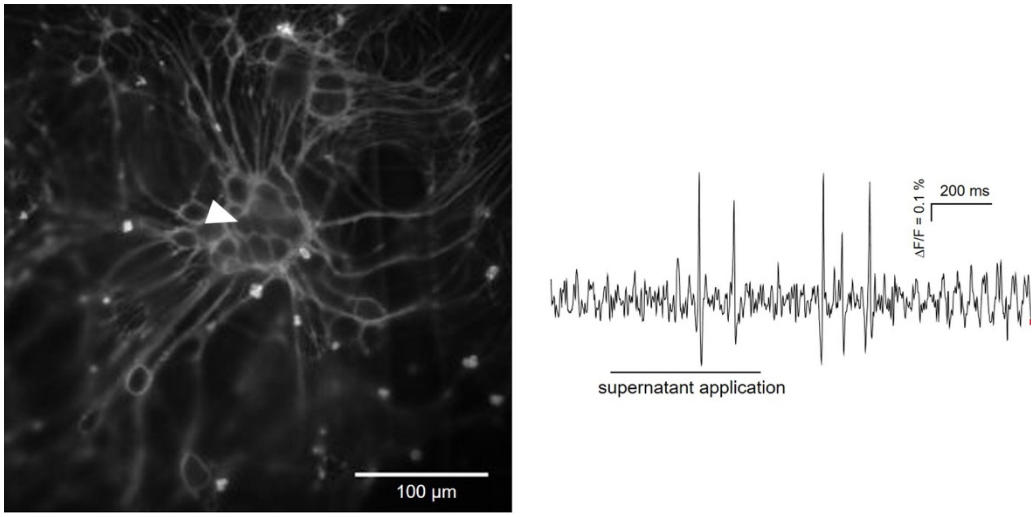

S2

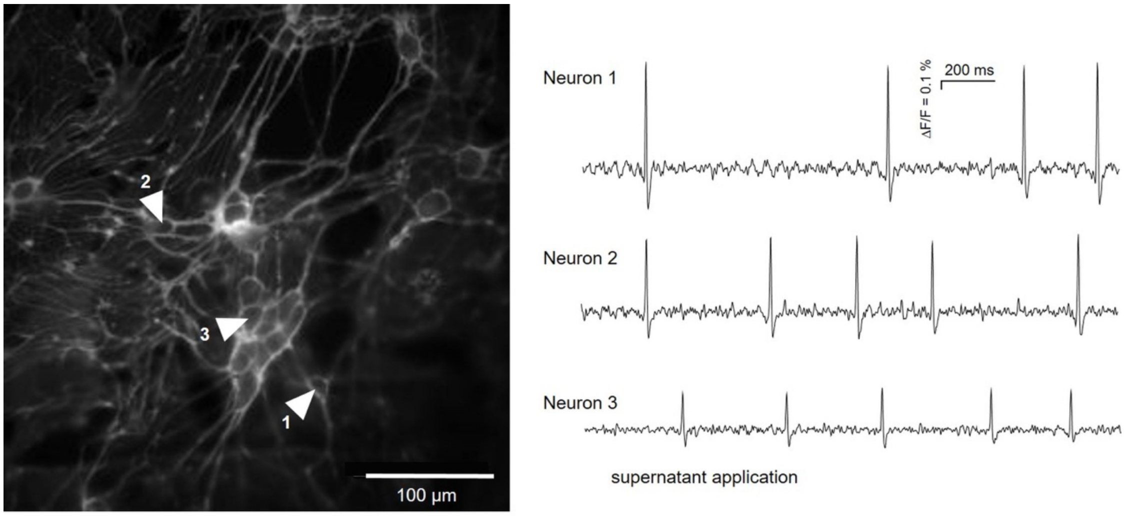

S3

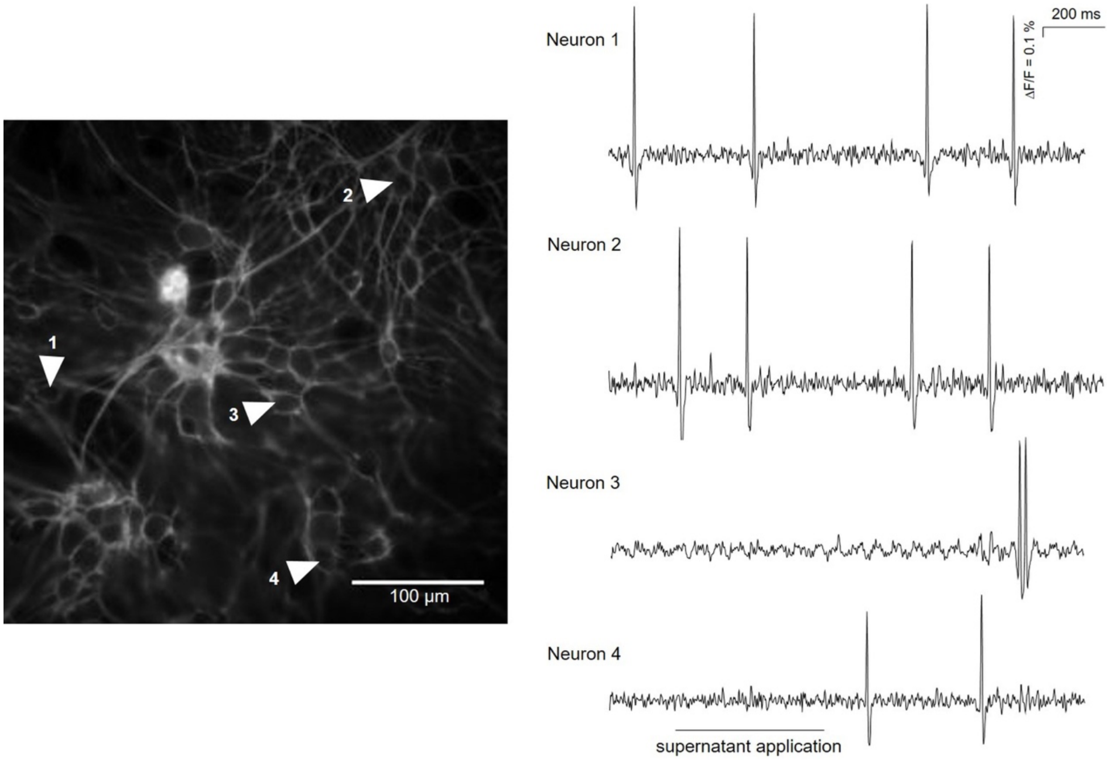

Figures S1 - S3. exemplarily show action potential discharge in guinea pig primary myenteric neurons, cultured for 12 days and stained by Di-8-ANEPPS, evoked by fecal supernatant application from a group A/control dog (Figure S1), a group C/PB responsive (Figure S2), and a group D/PB non-responsive dog (Figure S3).
